# Iodine Biofortification and Seaweed Extract-Based Biostimulant Supply Interactively Drive the Yield, Quality, and Functional Traits in Strawberry Fruits

**DOI:** 10.3390/plants12020245

**Published:** 2023-01-05

**Authors:** Beppe Benedetto Consentino, Lorena Vultaggio, Nicolò Iacuzzi, Salvatore La Bella, Claudio De Pasquale, Youssef Rouphael, Georgia Ntatsi, Giuseppe Virga, Leo Sabatino

**Affiliations:** 1 Department of Agricultural, Food and Forestry Sciences (SAAF), University of Palermo, Viale delle Scienze, Ed. 5, 90128 Palermo, Italy; 2 Department of Agricultural Sciences, University of Naples Federico II, 80055 Portici, Italy; 3 Laboratory of Vegetable Production, Department of Crop Science, Agricultural University of Athens, 11855 Athens, Greece; 4 Research Consortium for the Development of Innovative Agro-Environmental Systems (Corissia), Via della Libertà 203, 90143 Palermo, Italy

**Keywords:** biostimulants, algae extract, biofortification, stress tolerance, abiotic stress, *Fragaria* × *ananassa*

## Abstract

The horticultural sector is seeking innovative and sustainable agronomic practices which could lead to enhanced yield and product quality. Currently, plant biofortification is recognized as a valuable technique to improve microelement concentrations in plant tissues. Among trace elements, iodine (I) is an essential microelement for human nutrition. Concomitantly, the application of biostimulants may improve overall plant production and quality traits. With the above background in mind, an experiment was designed with the aim of assessing the interactive impact of a seaweed extract-based biostimulant (SwE) (0 mL L^−1^ (served as control) or 3 mL L^−1^ (optimal dosage)) and 0, 100, 300, or 600 mg L^−1^ I on the growth parameters, yield, fruit quality, minerals, and functional characteristics of the tunnel-grown “Savana” strawberry. SwE foliar application improved the plant growth-related traits, total and marketable yield, fruit color parameters, soluble solids content, nitrogen (N), potassium (K), and magnesium (Mg) fruit concentrations. Furthermore, an enhancement in the fruit dry matter content, ascorbic acid, and I concentration in fruits was detected when the SwE supply interacted with a mild I dose (100 or 300 mg L^−1^). The research underlined that combining SwE application and I biofortification increased the strawberry yield and quality and enhanced the plant nutritional status variation, thereby, determining a boosted strawberry I tolerance.

## 1. Introduction

Nowadays, agriculture is undertaking several efforts to enhance crop yield and quality, increase plant adaptability to climate change, and ameliorate plant tolerance to distresses [1,2]. Consequently, specific agronomic strategies are required [3,4] through the application of which all the above could be possible. Currently, as testified by Rouphael et al. [5], biostimulants represent an effective agricultural technique to elicit plant growth and development, as well as the yield and product quality of several vegetables. The advantages that arise through the application of biostimulants are mainly due to the plant physiological benefits and low application dosage per treatment [6,7].

Numerous authors [5,8,9,10,11,12,13,14] report that primary and secondary metabolism, nutrient element modulation, phytotoxic substance accumulation, and biotic and abiotic stress tolerance are affected by either microbial or non-microbial biostimulants. Among those substances, seaweed extracts (SwE) are an imperative class, and their benefits are connected to enzymes involved in nitrogen and carbon metabolic pathways, glycolysis, and the Krebs cycle. Moreover, SwE may stimulate mineral uptake and accumulation, as well as phytohormones biosynthesis [15,16,17]. Concurrently, there is growing attention to vegetable enrichment in functional components to elude human diseases, such as mineral malnutrition. Biofortification is considered an efficient and sustainable technique to increase microelement concentrations in vegetable crops [9,12,14]. Additionally, a biostimulant supply can interconnect with trace elements to augment the yield and quality of vegetables [12,14].

Iodine (I) is a crucial component of the human organism [18]. It is implicated in thyroid hormone synthesis, metabolic regulation processes, and the main physiological functions of the organism [19,20,21,22,23]. Low I intake causes goiter, reduced IQ, miscarriages, infant mortality, and birth defects [24]. The enrichment of fruits and vegetables with I could also be a valid alternative to the use of iodized salt in human nutrition. However, although this element is contained in foods, it is generally insufficient to provide the recommended daily intake (150–250 µg/day of I per adult) [25]. Moreover, as iodine is volatile, it frequently evaporates during storage, transportation, and cooking [26,27].

There are several reports on the I biofortification of leafy and fruiting vegetables [19,28,29,30,31], as well as of field crops [32,33]. However, few studies have been conducted on the I biofortification of strawberries.

*Fragaria* × *ananassa* is considered a relevant fruit crop in the northern hemisphere with a global production of over 8.861.381 tons estimated to worth over USD 22.065.163 million in 2020 [34]. China is the prominent world producer, followed by the USA and Egypt, while Spain and Russian Federation are the leading strawberry producers in Europe [34]. Strawberries are considered fruits of high preference by consumers; they are rich in bioactive components, such as anthocyanins, β-carotene, folate, phenolic compounds, vitamin C, vitamin E, and elements with strong relation to therapeutic benefits [35,36,37]. Considering that: (i) the application of SwE and agronomic biofortification with I are simple, safe, and sustainable strategies that result in the increased yield and quality of vegetables [38,39]; (ii) SwE can enhance plant abiotic stress tolerance [40,41], and (iii) as there are no reports concerning the interactive effects between SwE supply and I biofortification in strawberries, specific studies are required. Therefore, this research was designed to evaluate the influences of I and SwE foliar application on the plant growth parameters, yield traits, minerals, and nutritional and functional components of strawberries cultivated in tunnels.

## 2. Results

### 2.1. Plant Growth and Visual Quality

The statistics displayed that SwE and I and their interaction significantly affected plant growth parameters and plant visual quality (Table 1). The highest values of the No. of shoots, root collar diameter, and plant visual quality were collected from plots treated only with SwE. Plots treated with 3 mL L^−1^ of algae and 0 or 100 mg L^−1^ of I showed the highest plant height. The lowest plant height was recorded in non-biostimulated plants enriched with the highest I dose (Table 1).

### 2.2. Plant Yield

**Figure 1:**
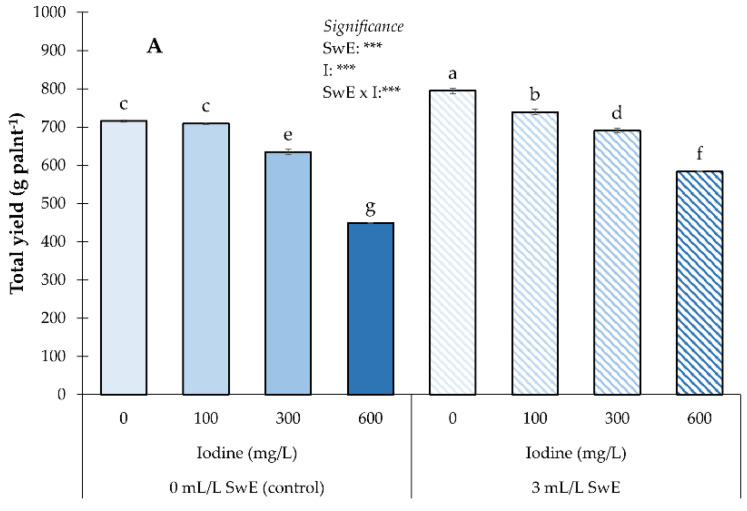

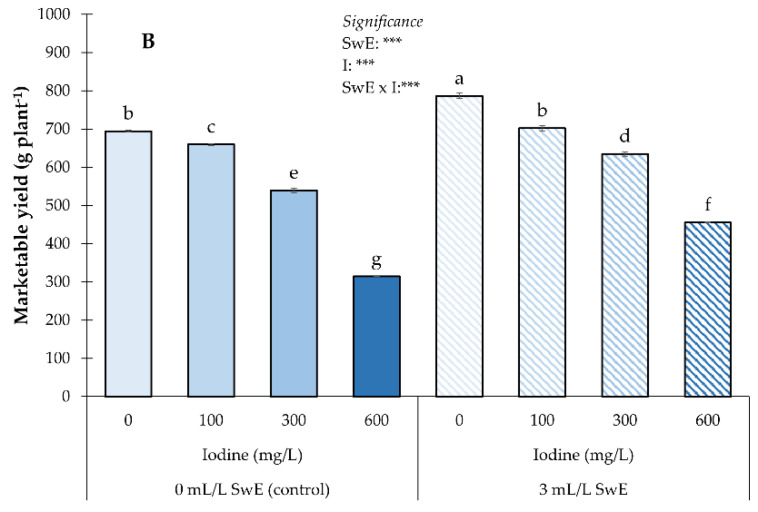
Influence of seaweed extract (SwE) and iodine (I) treatment on total yield (**A**) and marketable yield (**B**) of strawberries. Values with different letters indicate a significant difference at *p* ≤ 0.05. *** means significant at *p* ≤ 0.001. Bars represent mean ± SE.

Regarding yield traits (total yield and marketable yield), the statistics showed a significant impact of the experimental factors and their interaction. Plants biostimulated and not exposed to I treatment had the highest total and marketable yield (Figure 1A,B), whereas the lowest production performance was detected in plants treated with the maximum dose of biostimulant (600 mg I L^−1)^ and the non-biostimulated (Figure 1A,B). Overall, I biofortification negatively affected production traits, regardless of the SwE application.

### 2.3. Color Parameters

**Figure 2:**
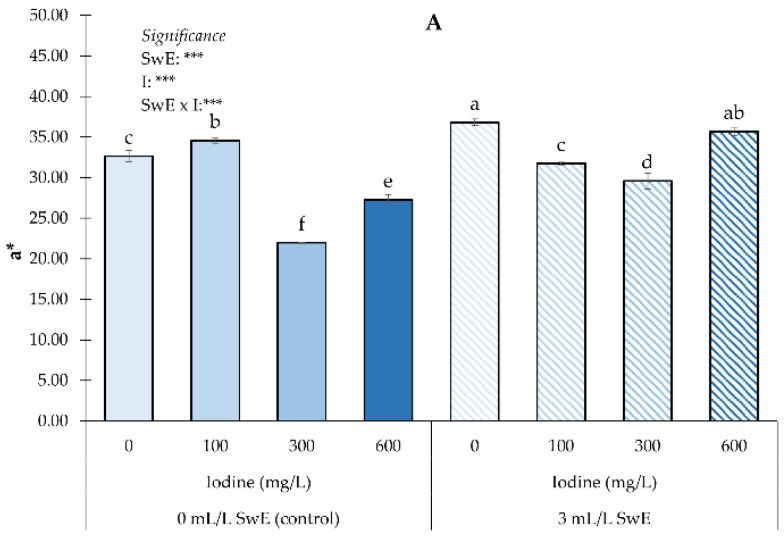

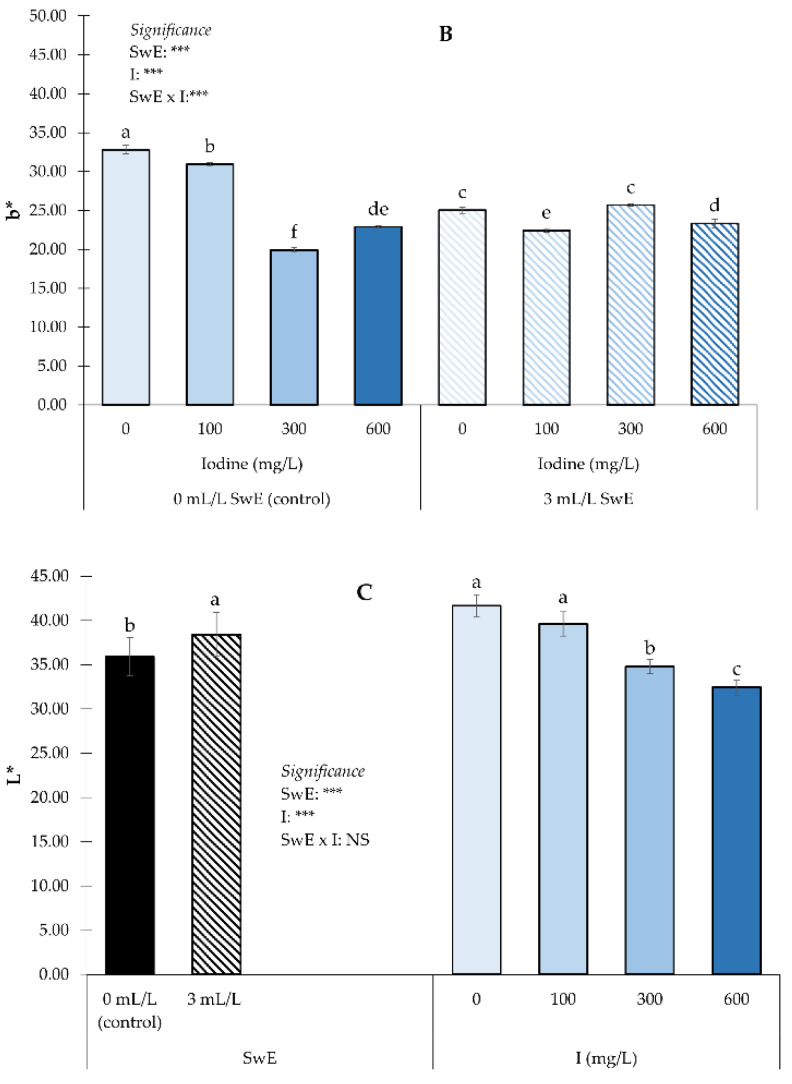
Influence of seaweed extract (SwE) and iodine (I) treatment on CIELab parameters—a* (**A**), b* (**B**), and L* (**C**)—of strawberries. Values with different letters indicate a significant difference at *p* ≤ 0.05. NS and *** means not significant or significant at *p* ≤ 0.001, respectively. Bars represent mean ± SE.

For two out of the three CIELAB color parameters (a* and b*), a SwE × I interaction was found (Figure 2A,B); in contrast, for the L* color parameter, no interaction was detected (Figure 2C). The highest value of a* was recorded in fruits from plots treated with the SwE and the non-biofortified, whereas the lowest a* were documented in fruits from non-biostimulated plants biofortified with 300 mg I L^−1^ (Figure 2A). The highest b* was found in the control plants, whereas the lowest was recorded in strawberries from non-biostimulated plots and those subjected to 300 mg of I per liter (Figure 2B). Concerning L*, regardless of the biofortification, fruits from the biostimulated plants showed a higher lightness than fruits from non-treated ones (Figure 2C). Averaged over the biostimulants, fruits from plants biofortified with a I dosage of 0 or 100 mg L^−1^ showed the highest values. Plants treated with the highest dose of I showed the lowest L* values (Figure 2C).

### 2.4. Fruit Dry Matter, Firmness, Soluble Solids Content, Ascorbic Acid, Phenolic Concentration, and Anthocyanins

**Figure 3:**
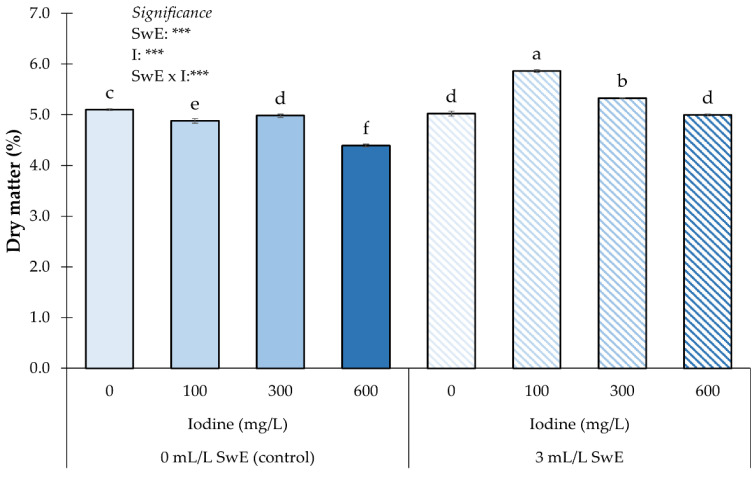
Influence of seaweed extract (SwE) and iodine (I) treatment on dry matter percentage of strawberries. Values with different letters indicate a significant difference at *p* ≤ 0.05. *** means significant at *p* ≤ 0.001. Bars represent mean ± SE.

Regarding the dry matter percentage, a significant influence of the SwE × I interaction was found (Figure 3). Fruits from plants treated with SwE and 100 mg I L^−1^ showed the highest dry matter percentage, followed by those from biostimulated plants and those subjected to 300 mg I L^−1^. The lowest dry matter percentage was recorded in fruit from non-biostimulated plants and those exposed to the highest I dose (Figure 3).

The ANOVA for firmness, soluble solid content (SSC), ascorbic acid, phenolic concentration, and anthocyanins showed a significant SwE × I interaction (Table 2). Data on firmness revealed that fruits from the control plots (0 mL L^−1^ of SwE × 0 mg I L^−1^) showed the highest values, whereas the SwE × 600 combination showed the lowest fruit firmness (Table 2). Regarding SSC, the fruits from biostimulated plants not exposed to the I biofortification showed the highest SSC (Table 2). The lowest SSC was detected in fruits from non-biostimulated plants treated with 600 mg I L^−1^. With regard to the ascorbic acid content, phenolic content, and anthocyanins, the highest results were always found in fruits from plants treated with 3 mL L^−1^ of SwE and 600 mg I L^−1^ (Table 2). The fruits from the control plants (control × 0) displayed the lowest ascorbic acid, phenolic, and anthocyanin concentrations (Table 2).

### 2.5. Fruit N and Mineral Profile

Regarding the mineral concentration (N, P, K, and Mg) of fruits (Table 3), the ANOVA showed a non-significant influence neither of the I biofortification nor of the SwE × I interaction (Table 3). Regardless of I biofortification, the SwE application increased N, K, and Mg fruit concentrations, without significantly affecting the concentration of P in the fruits (Table 3).

### 2.6. Fruit Iodine Concentration

**Figure 4:**
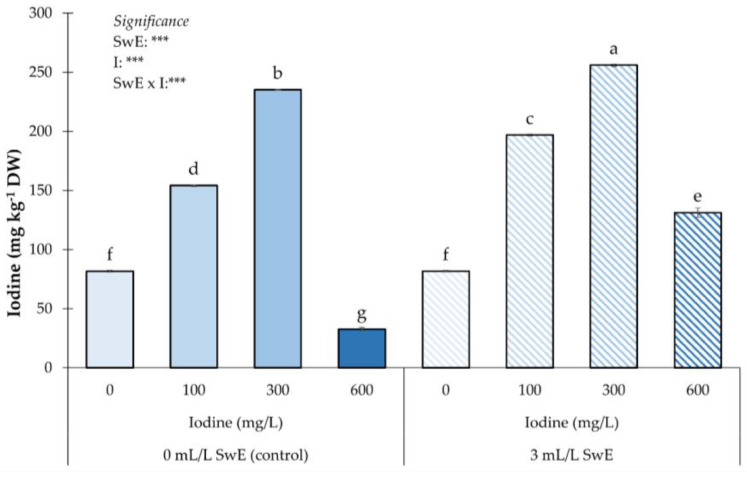
Influence of seaweed extract (SwE) and iodine (I) treatment on iodine concentration of strawberries. Values with different letters indicate a significant difference at *p* ≤ 0.05. *** means significant at *p* ≤ 0.001. Bars represent mean ± SE.

Regarding iodine, as shown in Figure 4, the ANOVA showed a significant difference for the SwE × I interaction. Fruits from the plants biostimulated with 300 mg of I L-1 accumulated the highest concentrations of I, followed by those from the control × 300 combination. The lowest concentration of I was recorded in non-biostimulated plants exposed to the highest dose of I (Figure 4).

### 2.7. Heat Map Analysis

**Figure 5:**
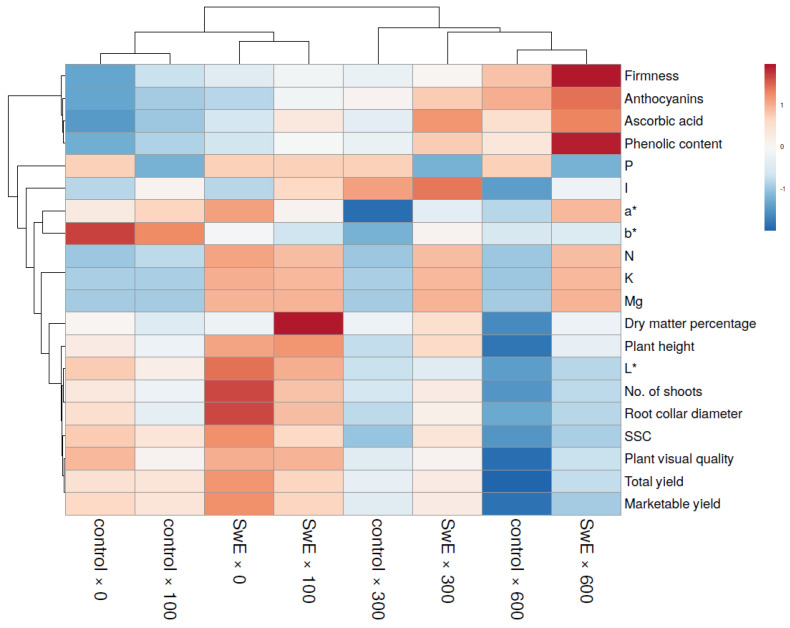
Analysis of all strawberry plant attributes using a heat map in response to treatment with seaweed extract (SwE) and iodine (I). The heat map was generated using the web tool ClustVis (https://biit.cs.ut.ee/clustvis/; accessed on 12 September 2022).

Figure 5 shows a heat map that graphically summarizes the effects of experimental factors on the strawberry plants The graphic analysis showed a dendrogram at the top (Dendrogram 1), including the treatments, and another on the left (Dendrogram 2), including the variables studied. Dendrogram 1 showed two main groups; the group on the left included the combinations with 0 and 100 mg I L^−1^, while the group on the right included the combinations with 300 and 600 mg I L^−1^ (Figure 5). In particular, the combinations not treated with SwE and biofortified with 0 or 100 mg I L^−1^ were divided from those treated with biostimulants and exposed to 0 or 100 mg I L^−1^. The latter combinations exhibited high firmness, anthocyanins, ascorbic acid, phenolic concentration, P, I, N, K, Mg, percentage of dry matter, plant height, L*, shoot number, root collar diameter, SSC, visual quality of the plant, total and marketable yield values. The group on the left included the control × 0 and control × 100 combinations. In this group, the combination control × 0 was characterized by the highest P, b*, percentage of dry matter, height of the plant, L*, number of shoots, root collar, SSC, visual quality of the plant, and marketable yield values. The group on the right included SwE × 0 and SwE × 100 combinations. In this group, the SwE × 0 combination stood out with the highest values of a*, N, L*, number of shoots, root collar diameter, SSC, total yield, and marketable yield. Analyzing the right side of Dendrogram 1, two main groups were identified. The group on the left included the control × 300 combination, while the group on the right included the SwE × 300, control × 600, and SwE × 600 combinations. The control × 300 was distinguished from the others by a lower firmness, anthocyanins, ascorbic acid, and a* and b* phenolic concentration. Within the left cluster, SwE × 300 was distinguished by higher I, b *, percentage of dry matter, plant height, L*, shoot number, root collar diameter, SSC, visual quality of the plant, and total and marketable yield. Looking at the right side of the latter cluster (comprising the control × 600 and SwE × 600 combinations), the control × 600 combination was characterized by lower firmness, anthocyanins, ascorbic acid, phenolic concentration, I, a*, b*, N, K, Mg, percentage of dry matter, plant height, L*, number of shoots, root collar, SSC, visual quality of the plant, and total and marketable yield.

## 3. Discussion

Modern consumers increasingly require food containing high amounts of macro- and micronutrients [1,42,43]. This need is linked to the lack of essential elements in human diets [23,44,45]. As a consequence, biofortification is recognized as a useful tool to enhance the concentration of trace elements, such as I [19,28,30], zinc [23,46,47], selenium [48,49,50,51], manganese [52,53,54], molybdenum [14,55,56,57,58], iron [59,60,61], and bioactive compounds in fruits and vegetables. Inadequate I intakes can create I deficiency disorders (IDD) in humans, with considerable consequences on life quality [62,63,64,65].

Considering that the application of algae extracts can improve mineral absorption and stress tolerance in plants [9,12,19], we evaluated the mutual effect of SwE foliar supply and I biofortification on the productive and qualitative features of strawberries grown under tunnels.

The outcomes of our study showed that I supply reduced plant yield. This is in contrast to the findings of Li et al. [1] who studied the effect of different forms of I on the growth and quality of strawberry plants, finding higher yields of strawberry plants treated with low doses of I. Our results on yield are in contrast to Lawson et al. [66] and Signore et al. [67], which found that the I supply had no significant effect on the yield of field-grown vegetables (kohlrabi, butterhead lettuce, and radish) and carrot, respectively. In contrast, our results are totally in agreement with the findings of Sabatino et al. [19] on curly endive. Consequently, we can assume that the effect of I on plant yield is a genotype-dependent trait and is strongly correlated with the I tolerance of the species. In previous studies [21,30,68,69], phytotoxic effects such as necrosis, chlorosis, and abscission of the leaves due to high I doses have been reported. In our study, combining SwE with high I doses (300 or 600 mg L^−1^) induced limited toxic effects. As reported by Blasco et al. [70], the utilization of a high quantity of I causes a slowdown of superoxide dismutase which is fundamental in preventing an oxygen reactive species (ROS) defense. Furthermore, as stated by Mynett and Wain [71], oxidation to elemental I, which occurs within cells, could cause adverse effects and inhibit photosynthesis.

The results highlighted that SwE application stimulates strawberry plant productivity. This is in line with the results of La Bella et al. [12], who obtained higher yields in spinach with the foliar application of SwE and those of Di Mola et al. [72] on lettuce and Rouphael et al. [73] on spinach, who observed an increase in yield when plants were subjected to SwE supply. Our results agree with those of Lawson et al. [66] who, by appraising the influence of I-based foliar treatments in lettuce and radish, found a significant decrease in yield compared to untreated plants. A comparable pattern of findings was described by Sabatino et al. [19] who, by investigating the effect of I-enrichment on curly endive, found that yields decrease as the I dose increases. These findings could be related to the harmful effect of I on photosynthesis, as I is stored, largely, in chloroplasts [28,31]. Alongside mitigating the influence of higher doses of I, SwE supply may elicit plant yield due to the polysaccharide content that stimulates endogenous hormonal homeostasis [73,74,75].

As regards the dry matter, the foliar application of SwE also limited the inhibitory effects of I; greater accumulation was obtained in biostimulated plants with 100 mg I L^−1^. Overall, these data fit with the findings of Incrocci et al. [76] who studied the I influence in different sweet basil genotypes cultivated in a hydroponic system, finding that a mild I dosage (10 μM KI) did not significantly affect plant dry matter. On the other hand, Rouphael et al. [73] stated an upsurge in the dry matter percentage of spinach treated with SwE.

In contrast with Budke et al. [21] who did not find significant variations in strawberry fruit firmness when administering various doses of I, in our study, fruit firmness was significantly affected by I doses; lower values were found in fruits from plants exposed to high iodine doses (300 or 600 mg I L^−1^). This can be explained because iodate (IO^_3_−^) is reduced to I^−^ which can disrupt plant membrane cells [62,66]. Our data also fit with the findings of Sabatino et al. [77] who evaluated the effect of grafting and different classes of biostimulants on eggplant, finding that fruits from biostimulated plants have higher firmness than those from control plants.

Our findings revealed a linear fruit SSC decrease as the I dose increased. Similar results were observed by Consentino et al. [31] who studied the combined effect between I biofortification and grafting in eggplant and found a reduced amount of fruit SSC in the biofortified plants. Concomitantly, SwE application enhanced the fruit SSC. These basic results are in agreement with previous studies showing that the application of different types of biostimulants in strawberries improve fruit SSC [38]. These results could be linked to the fact that—as reported by Nguyen-Quoc and Foyer [78]—SwE supply improves glucose biosynthesis, contributing to enhanced SSC.

In our study, the biostimulant supply and highest I dose elicited fruit functional trait (ascorbic acid, phenols, and anthocyanins) concentration. This agrees with the results obtained by various authors on strawberries [35,38]. Our findings also agree with the findings of Blasco et al. [70] who found an upsurge in ascorbic acid concentration in lettuce enriched with a high I dose. Sabatino et al. [19] reported a similar trend for ascorbic acid and phenols in curly endive plants treated with iodine. Moreover, Consentino et al. [31] reported that I-enriched fruits had the highest anthocyanin content. As stated by Medrano-Macías et al. [62], these findings are possibly connected to the fact that these secondary metabolites are enclosed in plant defense system stresses, embracing mineral stress. On the other hand, our findings showed that SwE treatments significantly increased strawberry functional traits. Similar outcomes were reported by Boselli et al. [79] who revealed an upsurge of phenol and anthocyanin concentrations in fruits treated with a plant-based biostimulant. However, Soppelsa et al. [38] found that SwE application enhances phenols and anthocyanin concentrations and reduces ascorbic acid in strawberry fruits. These data are similar to the findings of Rouphael et al. [73] who showed a higher phenol and anthocyanin concentration in spinach treated with SwE. The secondary metabolism stimulation (resulting in the increased biosynthesis of active compounds, such as phenols, anthocyanins, and ascorbic acid) could be related to the activity of a key enzyme (chalcone isomerase) involved in phytochemical homeostasis [80,81]. Our study pointed out that the mineral profile was not significantly affected by the different iodine doses; in contrast, the SwE treatments significantly increased N, K, and Mg concentrations in strawberry fruits. Several authors [19,31] discovered no significant variations in the mineral profile of I-biofortified vegetables. Our results agree with the findings of La Bella et al. [12] who observed higher concentrations of N, P, K, and Mg when administering SwE. Furthermore, Rouphael et al. [73] observed an increase in K and Mg concentrations in spinach plants treated with SwE. However, our results are not in agreement with those of Colla et al. [82] on tomatoes and Soppelsa et al. [38] on strawberries who observed no significant effect of SwE treatments on the mineral profile. We found that the fruit I concentration increased up to a dose of 300 mg I L^−1^. Concomitantly, the application of SwE improved the I content in all treated plants. This results are confirmed by various authors [19,29,70,76] who, carrying out I-based biofortification treatments on various species, found an increase in the element concentration up to an optimal dose above which the I content decreased (overdose). It has been observed that plants can absorb iodine from both epigeous and hypogeum organs [83]. Budke et al. [21] achieved an increase in iodine content, applying the element both through foliar treatments and soil applications. In a study on eggplant, Consentino et al. [31] obtained similar results. As noted by Lawson et al. [66], leaf treatments appear to improve iodine accumulation in plant tissues compared to radical administration.

## 4. Materials and Methods

### 4.1. Strawberry Materials and Trial Conditions

**Figure 6:**
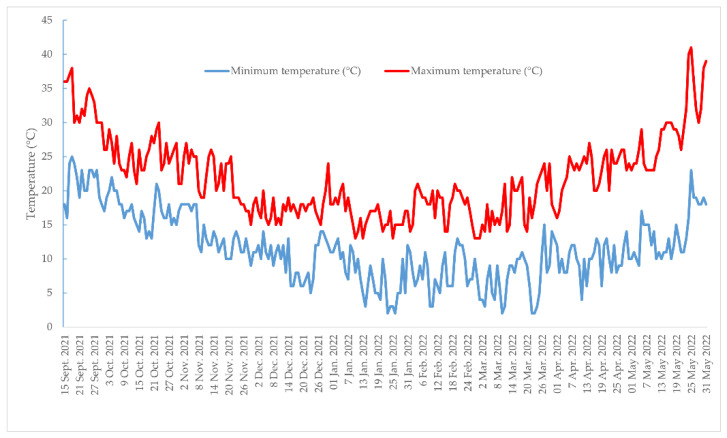
Maximum and minimum temperatures inside the tunnel during the strawberry growing cycle (from 15 September 2021 to 31 May 2022).

The experiment was performed throughout the year 2021–2022 growing period near Marsala, in an experimental field of the Department of Agricultural, Food, and Forest Sciences of Palermo (SAAF). Strawberry (*Fragaria* × *ananassa* “Savana”) plants were planted at a density of 8 plants m^−2^, following the traditional fall–winter–spring cultivation cycle conventionally adopted in Sicily [84]. The study was carried out under multiple tunnels covered with polyethylene. The soil hosting the experiment was solarized with polyethylene (0.05 mm) during the 2022 summer months (75 days of solarization in total). The film was kept for the terrain mulching. The soil originated from the conversion of the characteristically fertile Sicilian “sciare” soils (less than 80% of sand, 8.8% of limestone, rich in K2O, phosphorous, and nitrogen). Maximum and minimum temperatures values inside the tunnel were recorded (Figure 6).

### 4.2. Study Set-Up and Experimental Design

The biostimulant treatments were supplied to plants weekly starting seven days after transplanting using Kelpstar^®^ (Mugavero fertilizers, Palermo, Italy), an extract of *Ecklonia maxima* obtained via a cold micronization process, which prevents the degradation of seaweed bioactive components. They included organic N (1%), organic C (10%), hormones (11 mg L^−1^ of auxin and 0.03 mg L^−1^ of cytokinin), and organic substances (weight < 50 kDa) (30%). I was supplied via foliar spray using potassium iodate (purity 99.5%). I biofortification began 10 days after transplanting (DAT) and was repeated every 10 days. For each treatment, 0.5 L m^−2^ of solution was used. Two dosages of SwE (0 (as control treatment) or 3 mL L^−1^ (as recommended dosage)) were combined with four iodine (I) doses, namely, 0 (control), 100, 300, or 600 mg L^−1^. Each treatment included 3 replications, and each consisted of 15 plants arranged in a randomized complete block design (RCBD), for a total of 24 experimental units (2 SwE × 4 I × 3 replicates).

### 4.3. Measurements and Analysis

Marketable (fruits not affected by malformation or *Botrytis*) and total fruit yield were assessed on all plants from November to May (entire production cycle). The dry matter of fruit was obtained by placing 150 g of fruits at 105 °C to constant weigh, and its value was expressed as percentage.

At the first harvest (58 days after transplanting), strawberry growth parameters were evaluated. At harvest, the plant visual quality was also noted and recorded using a 9 to 1 scale where 9 is excellent, 7 is good, 5 is fair with marketable fruits, 3 is fair with unmarketable fruits, and 1 is seriously damaged.

Qualitative analyses were carried out on 10 fruit samples for each replication, belonging to the 4th harvest (150 days after transplanting). At the fruit sampling, plants received twenty SwE applications and fourteen I biofortification treatments. CIElab colour parameters were measured on the fruit with the use of a Chromameter CR-400 (Minolta Corporation, Ltd., Osaka, Japan). A penetrometer (Trsnc, Italy) was employed to measure fruit firmness. Values were shown as newtons (N). The soluble solids content (SSC) was appraised on filtered fruit juice with the use of MTD-045nD digital refractometer (Three-In-One Enterprises Co., Ltd., New Taipei, Taiwan).

### 4.4. Fruit Composition and Mineral Content

Ascorbic acid (AA) concentration of fruit was estimated with the Reflectometer RQflex10 Reflectoquant (Sigma-Aldrich, Saint Louis, MO, USA) and the Reflectoquant Ascorbic Acid Test strips. Findings were presented as mg AA 100 g^−1^ strawberry fresh weight.

The phenolic concentration of fruit was assessed following the method of Slinkard and Singleton [85]. Results were shown as mg 100^−1^ g dry weight (DW).

The anthocyanin concentration was measured as reported by Rabino and Mancinelli [86]. The anthocyanin concentration in fruit was expressed as mg of Cya-3-glucoside equivalent 100 g^−1^ of dry weight.

Fruit N concentration was appraised via the Kjeldahl method. The values were reported as g 100 g^−1^ DW. The P concentration was determined using the method of Fogg and Wilkinson [87]. The K concentration was assessed via atomic absorption spectroscopy. Magnesium concentration was determined as suggested by Morand and Gullo [88]. Minerals were expressed as mg g^−1^ DW.

The total I concentration in fruits was evaluated via ICP-MS, following the official methodology (European Standard BS EN 15111:2007). The I concentration was expressed as mg kg^−1^ DW.

### 4.5. Statistical Analysis and Heat Map

For statistics, the SPSS software package version 28 was used (StatSoft, Inc., Chicago, IL, USA). The influence of the treatments was appraised by two-way analysis of variance (ANOVA). Means were separated via the Tukey HSD test (*p* ≤ 0.05).

A color heat map analysis of all productive and qualitative aspects of strawberry plants in response to SwE applications and I supply was also produced by the web tool ClustVis https://biit.cs.ut.ee/clustvis/ (accessed on 12 September 2022).

## 5. Conclusions

Nowadays, improving nutraceutical profiles by maximizing fruit and vegetable yields and using eco-friendly tools is a major challenge for many researchers. Obtaining products with a high health value is one of the objectives to be pursued in order to augment the quality of life and improve the nourishing profile of diets.

In the current research, an *E. maxima*-based biostimulant application significantly elicited plant growth, yield, and nutritive and nutraceutical characteristics, as well as fruit mineral profiles. Concurrently, I-enrichment at the highest doses boosted the ascorbic acid, phenol, and anthocyanin concentrations. The highest fruit I concentration was observed by applying 300 mg L^−1^. Remarkably, our study highlighted that combining SwE application with I biofortification at a dosage of 100 mg^−1^ considerably mitigated the negative effects of I supply. Furthermore, these findings provide additional information on the interaction between I biofortification and sustainable agronomic practices, concluding that a reciprocal supply of SwE and I at 100 mg L^−1^ may proficiently mitigate yield reduction and, concomitantly, improve strawberry fruit quality.

## Figures and Tables

**Table 1:** Effect of seaweed extract (SwE) and iodine (I) treatment on plant height, No. of shoots, root collar diameter, and plant visual quality of strawberries.

Treatments		Plant Height (cm)		Shoot Number (plant^−1^)		Root Collar Diameter (mm)		Plant Visual Quality (1–9)	
SwE	Iodine (mg L^−1^)								
0 mL L^−1^ (control)	0	27.8	c	5.0	c	22.8	c	8.8	b
	100	25.9	d	4.5	d	20.2	e	7.0	c
	300	23.5	e	4.1	e	18.7	f	5.9	d
	600	18.9	f	3.2	f	17.2	g	2.5	f
3 mL L^−1^	0	31.3	a	6.3	a	26.4	a	9.0	a
	100	31.8	a	5.5	b	23.9	b	8.9	b
	300	29.3	b	4.9	c	21.7	d	7.0	c
	600	25.6	d	3.9	e	18.6	f	5.2	e
Significance									
SwE		***		***		***		***	
I		***		***		***		***	
SwE × I		***		*		***		***	

Values with diverse letters are significantly different at *p* ≤ 0.05. * and *** mean significant at *p* ≤ 0.05 or significant at 0.001, respectively.

**Table 2:** Influence of seaweed extract (SwE) and iodine (I) treatment on firmness, soluble solid content (SSC), ascorbic acid, phenolic concentration, and anthocyanins of strawberries.

Treatments		Firmness (N)		SSC (°Brix)		Ascorbic Acid (mg L^−1^)		Phenolic Concentration (mg 100 g^−1^)		Anthocyanins (mg Cya-3-Glucoside 100 g^−1^)	
SwE	Iodine (mg L^−1^)										
0 mL L^−1^ (control)	0	−7.52	f	7.0	b	40.6	h	443.0	h	85.9	h
	100	−7.05	e	6.7	c	45.6	g	455.0	g	91.5	g
	300	−6.77	d	5.5	d	52.2	e	474.3	e	107.0	d
	600	−6.11	b	5.1	e	61.0	c	491.3	c	120.8	b
3 mL L^−1^	0	−6.93	e	7.4	a	49.8	f	462.3	f	93.4	f
	100	−6.72	cd	6.9	b	59.3	d	480.3	d	104.0	e
	300	−6.59	c	6.7	c	68.2	b	503.3	b	117.6	c
	600	−5.45	a	5.6	d	69.4	a	538.3	a	127.5	a
Significance											
SwE		***		***		***		***		***	
I		***		***		***		***		***	
SwE × I		**		***		***		***		***	

Values with diverse letters are significantly different at *p* ≤ 0.05. ** and *** mean significant at *p* ≤ 0.005 or significant at 0.001, respectively.

**Table 3:** Influence of seaweed extract (SwE) and iodine (I) treatment on mineral concentrations (N, P, K, and Mg) of strawberries.

Treatments	N (g 100 g^−1^ DW)		P (mg g^−1^ DW)		K (mg g^−1^ DW)		Mg (mg g^−1^ DW)	
SwE								
0 mL L^−1^ (Control)	8.61	b	3.46		6.46	b	1.68	b
3 mL L^−1^	9.61	a	3.45		8.72	a	1.96	a
Iodine (mg L^−1^)								
0	9.13		3.46		7.65		1.83	
100	9.13		3.46		7.58		1.83	
300	9.08		3.46		7.58		1.80	
600	9.08		3.46		7.55		1.81	
Significance								
SwE	***		NS		***		***	
I	NS		NS		NS		NS	
SwE × I	NS		NS		NS		NS	

Values with different letters indicate a significant difference at *p* ≤ 0.05. NS and *** mean not significant at *p* ≤ 0.005 or significant at 0.001, respectively.

## Data Availability

Not applicable.
